# Miscellanies

**Published:** 1837-07-01

**Authors:** 


### MISCELLANIES.

### Irish Medical Charities Bill.

Concurrently with a Bill for the Relief of the Destitute Poor in Ireland, the Government has introduced one for the regulation of the medical charities of that country—a measure as humane as it is wise and necessary.

This is a subject of considerable importance, and we are anxious to bring it under the consideration of the profession in each country.

It may not be known to many on this side of the Channel, that the mode of raising funds to supply the sick poor of Ireland with medical aid, is very different from that resorted to in England. In the latter country, all the hospitals and dispensaries are supported by private contributions—parish medical aid alone being supplied from a public fund. But this fund is raised as a portion of a poor-rate. In Ireland, on the contrary, all the metropolitan and county infirmaries, all the fever hospitals, dispensaries, and lunatic asylums are supported either entirely or partly, but chiefly by public funds, raised from each county as a part of the county-rates.

The sum thus expended on the public medical charities of Ireland is about £180,000. a year; and the number of sick poor receiving medical aid in them or from them is, we are informed by persons well conversant with the subject, no less than 1,360,000 annually. But, from a variety of circumstances, the existing institutions are quite inadequate to afford professional attendance to that portion of the community *really* requiring it, whilst many who are not fit objects are enabled to throw themselves on these charities, thus depriving the sick paupers of a portion of the funds which should be exclusively reserved for them, encreasing the public assessment unnecessarily, and depriving the profession of any advantages they might otherwise obtain from attendance on the class alluded to.

These are not mere surmises or assertions; that such is the fact has been shewn beyond all doubt or controversy. The credit of having first pointed out the defects and abuses of the medical charities of Ireland is due to Mr. Phelan of Clonmell, whose statistical enquiry we have before given some account of. His exposure of the defects of medical charities of his country, and the remedial measures which he suggests for their improvement have been fully agreed to by the four medical men who inspected a great many of these institutions under the Irish Poor Enquiry Commission.—(*See Appendix*, B.) And these suggestions have been made a considerable part of the ground-work of the measures proposed for the relief of the destitute poor of Ireland by the Commissioners.—*(See Third Report Irish Poor Enquiry Commission.)*

Under these circumstances, when the Government was about to provide for the destitute poor of Ireland, it was but right that it should also provide a better system of affording the sick poor the necessary medical aid—there being so intimate a connexion between destitution and sickness, which, in fact, are often only convertible terms.

Lord Morpeth has accordingly introduced "A Bill for the better Regulation of Hospitals, Dispensaries, and other Medical Charities in Ireland." Without entering into the details of the Bill, we will state its chief objects; which are first, to repeal the various acts which empower Grand Juries to make presentments for the different medical charities, *and which they can only do under certain circumstances*; 2dly, to authorize the Lord Lieutenant to appoint four physicians or surgeons as inspectors of public medical charities in Ireland, which inspectors are to keep an office, and to form a Board, and are empowered to make rules and regulations for the general management of each class of these charities; and who are also required to inspect each institution twice a year, to make a report respecting it, to receive half-yearly returns of the state of disease in each district, and to submit an abstract of the whole to Parliament every year. The third portion of the Bill requires of the local governors, or committee of any hospital, dispensary, &c. to send a yearly estimate of the amount of funds which will probably be necessary for the ensuing year, with an account in detail of the expenditure, &c. for the past years to the medical inspectors, on whose examination, and, of course, on whose recommendation the Lord Lieutenant is authorized to advance the *necessary* sum to such local committee, to be by them expended under such regulations as the medical inspectors, *sanctioned by the Lord Lieutenant*, may frame for the purpose. And the sums so advanced, it is imperative on the county fiscal authorities to grant, for the purpose of repaying the Government.

In addition, the Poor Law Commissioners are to have an *inspectorial* power over all these public charities, and are authorized to make such regulations for their government as may insure that they shall be managed according to the existing wills, statutes, laws, &c. of each, or of each class. The object of this appears to be, to provide that the medical inspectors shall make their regulations in conformity with the existing laws, &c. But the Bill wisely secures the assistance of professional men, to make the necessary rules and regulations for the general medical managements of all the public instiutions of the country;—a provision which must, in a great measure, obviate the difficulties, or the jealousies, that would be sure to arise, were the Poor Law Commissioners the only authority to regulate such numerous and such expensive charities, whose good management is essentially necessary for all classes of the people of Ireland.

Hitherto the infirmary, and much of the fever hospital, accommodation of Ireland, was what may be called *too much concentrated*; and on that account less useful to the sick poor beyond a certain distance. This Act provides for the erection of a greater number of hospitals, &c. under certain limitations—thereby enabling the sick poor of every district to obtain medical aid. This is a vast advantage; for how can it be expected that many bad surgical or acute medical cases will bear removal to a hospital 15, 20, or 40 miles distant, as is now necessary. Again, it will provide dispensary aid in each district, and at a very moderate expense, by locating an educated medical man at a fair salary. But, in addition, the appointment of medical inspectors, (for, we are sure the Government will appoint none but men of character, and of a matured judgment,) will ensure that the public and private funds shall be judiciously and faithfully disbursed—and that the really sick poor, and those only, shall be provided for.

We request of the medical profession in this country to take a lesson from the active exertions of their Irish *confreres*, particularly of those of Munster, in this matter. They have induced the Government to admit and adopt a principle which we most highly approve of—that medical men should be the immediate and directing parties to regulate the public medical charities of a country. It is quite evident, from the account here given of the different means by which funds are raised for hospitals, &c. in this country and in Ireland, that such a machinery is much more called for in the latter country than in the former; but still we cannot help observing, that when so large a sum as between 2 and £300,000. a year is expended on gratuitous medical parish aid in England, even here some medical functionaries would be useful in framing rules, &c. for the expenditure of so much money, so that the profession and the public might have the advantage of those who must be most conversant with the subject.

We wish the Irish Medical Charities' Bill every success, and trust the Legislature—those of all parties—will pass it into law.

We cannot conclude without expressing how deeply the profession and the public are indebted to Dr. Nugent, of Cork, and Surgeon Phelan, of Clonmell, for their exertions in this measure. They have left their professional avocations in Ireland, and have been in London for several months, offering such suggestions as great attention to the subject has enabled them to do with advantage, and watching over the interests of the sick poor and the medical practitioner of the Sister Isle. We sincerely hope that such exertions and sacrifices will not be allowed to go unrewarded, and that those with whom the patronage lies, will not fail to place these gentlemen in situations where their zeal and abilities may continue and complete the arrangements they have so successfully organized.

